# RFID Ultra-High Frequency Tag Antenna Based on SRR Resonant Superstrate

**DOI:** 10.3390/s26041233

**Published:** 2026-02-13

**Authors:** Zhenhao Huang, Minghan Ke, Haonan Zhang, Lihao Luo, Chaohai Zhang, Guozhi Zhang

**Affiliations:** 1 Hubei Engineering Research Center for Safety Monitoring of New Energy and Power Grid Equipment, Hubei University of Technology, Wuhan 430068, China; 13018053772@163.com (Z.H.); 13387560942@163.com (M.K.); 13035139268@163.com (H.Z.); m18102336261@163.com (L.L.); 2 School of Automation, Nanjing University of Aeronautics and Astronautics, Nanjing 211106, China; 13626513732@163.com

**Keywords:** SRR, RFID, antenna, ultra-high frequency, regulation and control

## Abstract

Addressing the pressing need to extend the communication range of RF RFID tag antennas, this paper introduces a novel UHF RFID tag antenna technology based on resonant superstrate regulation using a Split-Ring Resonator (SRR). First, a finite element model of the UHF RFID folded dipole antenna was constructed based on the tag chip’s port impedance. Subsequently, a Two-element SRR resonant superstrate was employed to enhance the dipole antenna’s gain through “resonance and near-field coupling” technology. A folded dipole antenna gain-enhancing SRR resonant superstrate unit was designed, and a multi-parameter joint optimization method was adopted to obtain the optimal SRR resonant superstrate configuration for regulating the dipole antenna. Near-field coupling technology was used to design SRR resonant superstrate elements that enhance the folded dipole antenna’s gain. A multi-parameter joint optimization method was employed to obtain the optimal structural parameter set for the SRR resonant superstrate-controlled dipole antenna. Finally, simulations and experimental measurements of the RFID antenna performance revealed that: within the 920–925 MHz band, the maximum measured forward reading distance enhancement reached 62.1%. The research findings significantly enhance the practical performance of UHF RFID tags in complex environments, enabling more stable and efficient long-range identification in applications such as logistics tracking, asset management, and smart warehousing.

## 1. Introduction

The recent rise of the Internet of Things has accelerated the development of RFID communication technology and antenna sensing technology. As a core component of the IoT perception layer, RFID tags play a crucial role in the informatization and intelligent development of industries such as logistics tracking, smart warehousing, and asset management. Additionally, due to their wireless, low-power, and low-cost characteristics, RFID tags are an essential means for implementing wireless sensor network technology [1]. A typical RFID system primarily consists of three components: a computer control terminal, an RFID reader/writer (including an antenna), and tags [2].

**Figure 1:**
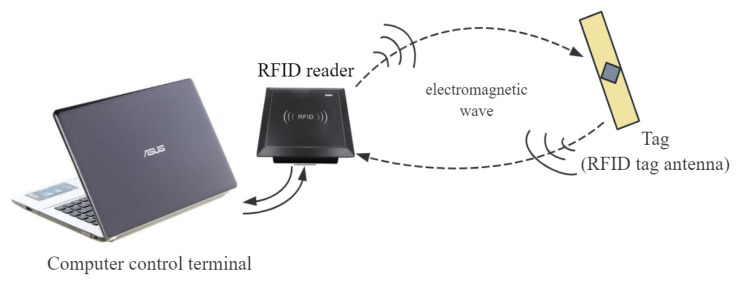
Working diagram of the RFID system.

The operation of an RFID system is illustrated in Figure 1. However, existing RFID tag antennas commonly suffer from reduced radiation efficiency and gain due to miniaturization demands, resulting in insufficient communication range. This has become a prominent bottleneck limiting their large-scale application in complex scenarios [3].

In RFID systems, tag antennas receive and transmit signals, and their performance directly impacts read range. Enhancing RFID tag antenna gain primarily involves two approaches: Firstly, optimizing existing antenna structures—for instance, Reference [4] employs evolutionary algorithms to refine structural parameters of folded dipole antennas, thereby increasing antenna gain; in Reference [5], by adjusting the T-type impedance matching structure and optimizing the antenna impedance, the antenna impedance is better matched with the chip impedance, thereby further enhancing the antenna gain. However, such methods are rather cumbersome and can only be used to bend dipole antennas. Secondly, loading special structures: For instance, loading finger-shaped structures to increase the electrical length of the antenna or loading double-layer serpentine structures to endow the RFID tag antenna with anti-metal properties, thereby enhancing the reading distance of the antenna in a metallic environment. However, when applied to other environments, there is no significant improvement effect, which has certain limitations [6,7]. These methods generally have the disadvantages of high cost, complex structure, and weak universality.

Based on this, this paper proposes UHF RFID tag antenna technology regulated by a Two-element SRR resonant superstrate. First, a finite element model of the UHF RFID folded dipole antenna was constructed based on the port impedance of the tag chip. Then, utilizing the “resonance and near-field coupling” technology of the SRR resonant superstrate, a folded dipole antenna gain enhancement SRR resonant superstrate unit was designed to achieve significant local magnetic field enhancement and near-field coupling optimization within the operating frequency band, thereby improving the performance of the SRR resonant superstrate unit. Near-field coupling technology was used to design SRR resonant superstrate elements that enhance the gain of the folded dipole antenna. This achieves significant local magnetic field enhancement and near-field coupling optimization within the operating frequency band, thereby improving the radiation gain and directivity of the RFID tag antenna. A multi-parameter joint optimization method was employed to obtain the optimal structural parameter set for the SRR resonant superstrate-controlled dipole antenna. Finally, prototype RFID antennas and SRR resonant superstrate were fabricated, and their performance was experimentally evaluated.

## 2. Modeling and Simulation of RFID Tag Antennas

RFID tag antennas are categorized by operating frequency bands: low-frequency (LF), high-frequency (HF), and ultra-high-frequency (UHF). LF RFID operates at 120–130 kHz; HF RFID at 13.56 MHz; and UHF RFID at 860–960 MHz [8]. Among these, UHF RFID antennas offer relatively longer coverage distances, typically ranging from tens of centimeters to several meters, making them suitable for scenarios such as warehousing and logistics tracking [9,10,11]. Common UHF RFID tag antennas include dipole antennas [12,13], microstrip antennas [14], and fractal antennas [15]. Dipole antennas are widely adopted due to their simple structure and low cost. However, the gain of these miniaturized antennas ranges from 0 to 2 dB, with typical read ranges of 1.5 to 2 m—a distance that is too short and requires further enhancement.

### 2.1. Design of Ultra-High Frequency Folded Dipole Tag Antenna

The dipole antenna features a relatively simple structure, consisting of a pair of symmetrically placed conductors with a center feed configuration. Assuming its center frequency is 915 MHz, we can estimate it using Formula (1):

(1) $$L=\frac{\lambda }{4}=\frac{c}{4\;\sqrt{\varepsilon \;}f}$$

Here, *ε* is the relative dielectric constant of the dielectric, *c* is the propagation speed of the electromagnetic wave, and *f* is the operating frequency. It is calculated that the corresponding single-arm length *L* of the dipole antenna in vacuum needs to reach 82 mm. Clearly, this does not meet the requirement of miniaturization of the tag antenna. Therefore, to achieve the miniaturization of the antenna, we can bend the antenna arm and increase the number of bends to realize the miniaturization of the tag antenna [16,17].

**Figure 2:**
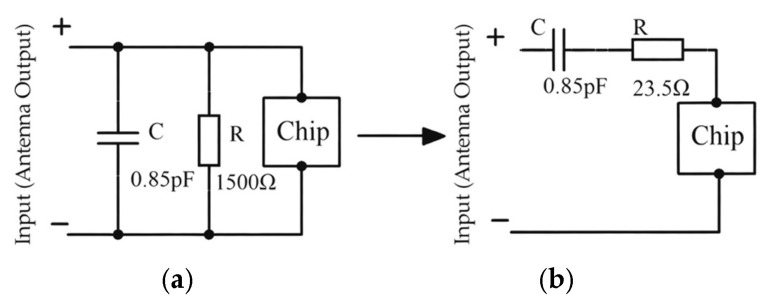
(**a**) The equivalent parallel RC circuit diagram; (**b**) the equivalent series RC circuit diagram.

For the folded dipole RFID tag antenna, the Alien Higgs-3 chip produced by Alien Technology in San Jose, California, United States is employed [18]. Its equivalent parallel input impedance is 1500 Ω, coupled with an equivalent capacitance of 0.85 pF. The chip impedance is a parallel RC structure, as shown in Figure 2a. To match the antenna, it must be converted into a series model (Figure 2b). Calculations yield the chip impedance as 23.5–204.1 jΩ. The port impedance is finally set to the conjugate impedance of the chip impedance as 23.5 + 204.1 jΩ. Additionally, the antenna substrate employs flexible material (FPC) with dimensions minimized as much as possible.

### 2.2. Antenna Optimization and Performance Analysis

**Figure 3:**
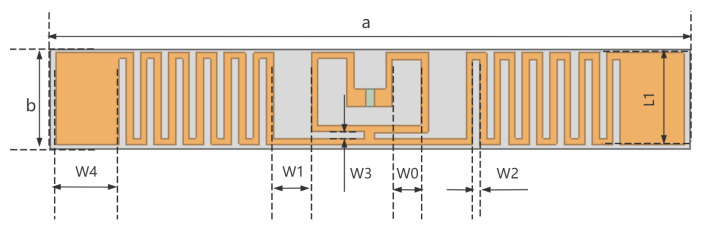
Dimension marking related to antennas.

**Figure 4:**
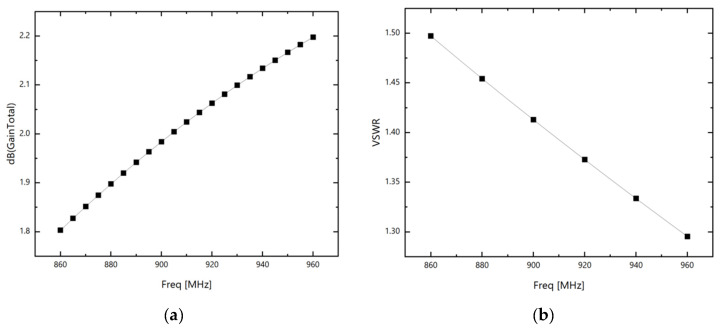
(**a**) The gain of RFID dipole tag antennas; (**b**) the standing wave ratio of the RFID dipole tag antenna.

Reference [19] proposed a common RFID folded dipole tag antenna structure achieving a maximum gain of 1.8 dB. However, due to its large size and rigid FR4 substrate material, this paper attempts to replace the substrate with flexible FPC, modify its thickness, and further optimize the antenna arms and coupling loop. The preliminary structure obtained is as follows: The antenna length *a* of the antenna is 71 mm, the width *b* is 11 mm, and the thickness *h* is 0.13 mm. The line width *W* is 0.725 mm, with 11 bends on a single arm. The bending interval *W*_2_ is 0.83 mm, and the length *L*_1_ of each bend is 8.8 mm. The power supply mode is central power supply, and the port impedance is set at 23.5 + 204.1 jΩ. The antenna model is shown in Figure 3:

The parameters of the RFID dipole tag antenna after joint optimization with multi-size parameters are shown in Table 1:

After the modeling was completed in the HFSS electromagnetic software (version 2024), the gain and standing wave of the antenna were obtained through simulation, as shown in Figure 4:

As shown, due to miniaturization requirements, the gain of RFID tag antennas is generally not very high. To enhance antenna gain, this paper further improves it by loading an SRR structure.

## 3. Two-Element SRR Resonant Superstrate Control of Folded Dipole Tag Antennas

### 3.1. The Principle of SRR Resonant Superstrate Regulation to Enhance Antenna Gain

SRR consists of two concentric rings with symmetrical slots of different radii, first proposed by Pendry et al. in 1999. When a clockwise current flows in the inner ring, the magnetic field direction inside the inner ring points toward the plane of the paper. In the region immediately adjacent to the inner ring on the outer side, the magnetic field direction also points toward the plane of the paper. The outer ring induces a counter-magnetic field to oppose the change in the inner ring’s magnetic field, generating an induced magnetic field directed outward (away from the plane of the paper) within the outer ring. This corresponds to an induced counterclockwise current in the outer ring. At this point, the integrated system formed by the inner and outer rings can be equivalently modeled as a “magnetic dipole,” capable of coupling with the original external magnetic field [20].

When the external magnetic field frequency approaches the SRR’s resonant frequency, the SRR exhibits “negative magnetic permeability”: in the resonant state, the polarization direction of the SRR’s equivalent magnetic dipole opposes the incident external magnetic field (resulting in negative magnetic susceptibility). Consequently, its magnetic response direction is entirely opposite to that of conventional paramagnetic/diamagnetic materials, significantly canceling or even reversing the distribution of the incident magnetic field. Simultaneously, SRRs exhibit electric field coupling—when excited by a time-varying electric field parallel to the ring plane, they can synergistically modulate electromagnetic waves with negative permittivity [21]. These properties enhance electromagnetic wave intensity through the following mechanisms:

1. Reverse polarization cancels interference: When SRRs resonate, their equivalent magnetic dipole polarization opposes the incident external magnetic field (negative magnetic permeability), canceling or reversing the distribution of interfering fields. This reduces unwanted magnetic field effects, thereby amplifying the effective electromagnetic wave intensity [22];
2. When the operating frequency approaches the SRR’s resonance frequency, the SRR generates intense localized magnetic and electric fields on its rear surface. The interaction between the SRR and the electromagnetic field behind the antenna alters the field distribution in the rear region. This redistributes energy toward the original antenna, facilitating coupling of rear-area energy into the main antenna’s radiation path rather than dissipating it as loss or radiating ineffective power.

Therefore, loading an SRR structure onto an RFID tag antenna can leverage its physical properties to enhance antenna gain [23,24].

When the SRR is cut along a direction perpendicular to the aperture, the separated halves can each be treated as equivalent to a capacitor, and these two capacitors *C*_1_ and *C*_2_ form a series capacitor circuit, while the metal part is regarded as the inductor *L*_1_.

Thus, the entire SRR can be equivalent to an LC resonant circuit:

(2) $$f=\frac{1}{2\pi \sqrt{\;L_{1}{\;C}_{0}}}$$

Suppose *C*_3_ is the equivalent capacitance of the ring, *C*_1_ = *C*_2_ = *C*_3_/2. In the formula, if *C*_0_ is the series capacitance formed by the capacitance of the upper and lower parts, then *C*_0_ = *C*_3_/4. Thus, the SRR size operating at the working frequency of the UHF RFID tag antenna can also be calculated from it [25,26,27].

### 3.2. The Design of Two-Element SRR Resonant Superstrate Units

**Figure 5:**
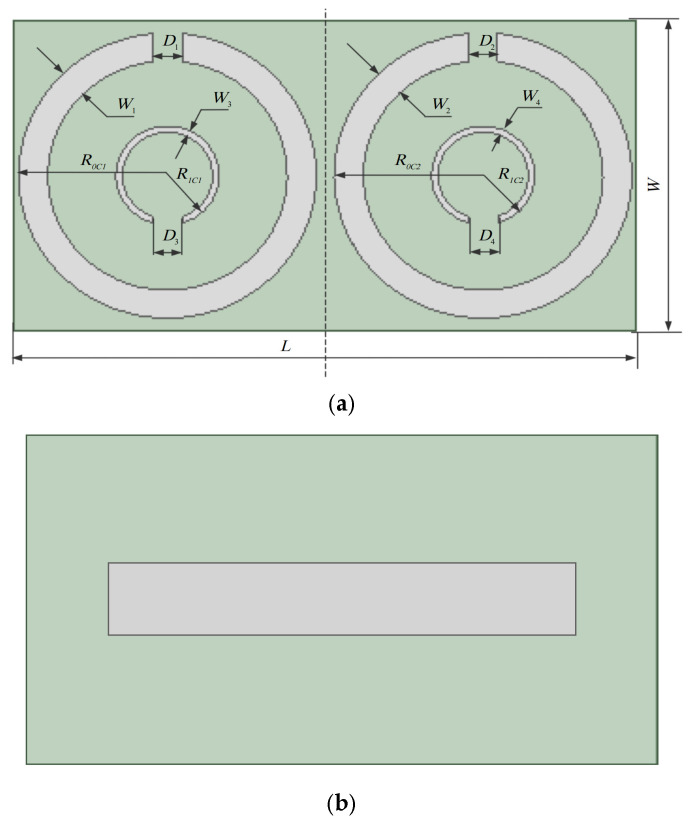
SRR unit model diagram. (**a**) Front-side model of SRR unit; (**b**) SRR unit reverse model (The gray rectangle in the reverse model solely indicates the positional relationship between the SRR and the RFID tag antenna. The RFID tag antenna is directly affixed to the gray area on the back of the SRR substrate; the gray rectangle serves no other purpose).

**Figure 6:**
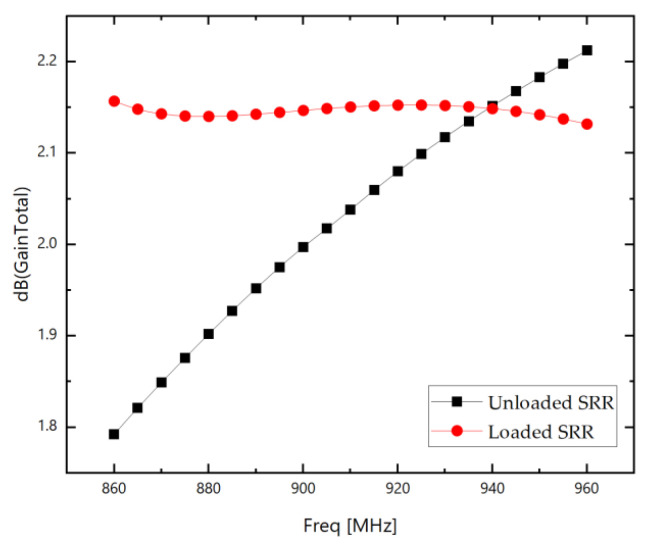
Comparison chart of gain before and after loading the SRR resonant superstrate unit.

The SRR unit uses a flexible FPC with a relative dielectric constant ε_r_ of 3.5 as the dielectric substrate. Its length *L* is 96 mm, width *W* is 50 mm, and thickness *h* is 0.183 mm. The front side of the dielectric substrate is composed of two SRR patches with the same opening direction. The size of the SRR unit model with a central operating frequency of 915 MHZ can be calculated through the formula, as shown in Figure 5:

The initial parameters of the two SRRs are the same. Suppose the SRR on the left side of the figure is *C*_1_ and that on the right side is *C*_2_, the outer ring radii *R*_0*C*1_ and *R*_0*C*2_ are both 23 mm, the inner ring radii *R*_1*C*1_ and *R*_1*C*2_ are both 7.5 mm, the distances *L*_1_ and *L*_2_ from *C*_1_ and *C*_2_ to the central axis are both 1.35 mm, the outer ring widths *W*_1_ and *W*_2_ of *C*_1_ and *C*_2_ are both 7 mm, and the outer ring openings *D*_1_ and *D*_2_ of *C*_1_ and *C*_2_ are both 4.5 mm. The inner ring widths *W*_3_ and *W*_4_ of *C*_1_ and *C*_2_ are both 1 mm, and the inner ring openings *D*_3_ and *D*_4_ of *C*_1_ and *C*_2_ are both 4.5 mm (Note: The gray rectangle in the reverse model solely indicates the positional relationship between the SRR and the RFID tag antenna. The RFID tag antenna is directly affixed to the gray area on the back of the SRR substrate; the gray rectangle serves no other purpose). The size parameters of SRR are shown in Table 2 as follows:

Figure 6 shows the gain comparison diagram of the RFID antenna before and after loading the Two-element SRR resonant superstrate unit, simulated using Ansoft HFSS software (version 2024):

### 3.3. Optimization of SRR Unit Size Parameters

**Figure 7:**
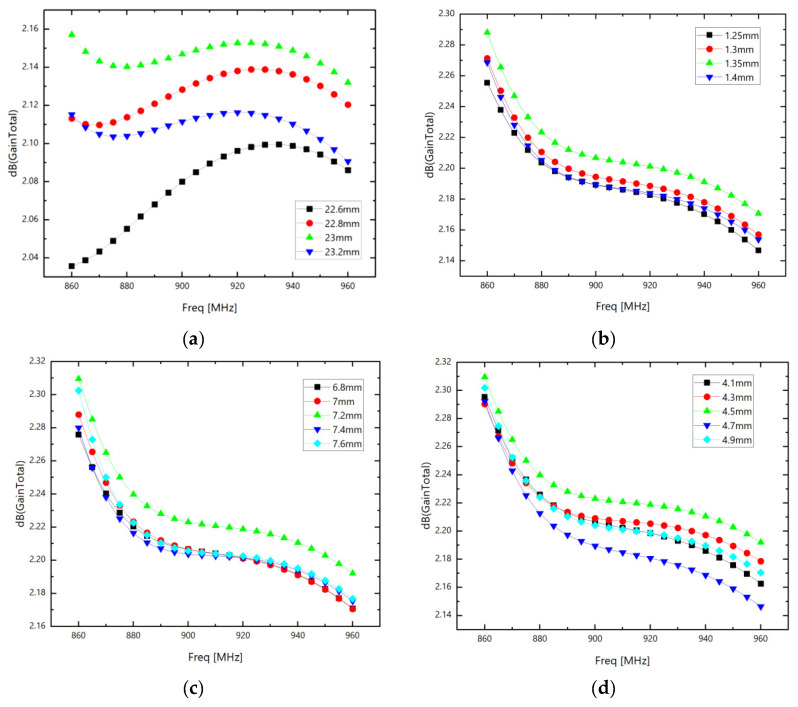
(**a**) Parameter optimization of *R*_0*C*1_; (**b**) parameter optimization of *L*_1_; (**c**) parameter optimization of *W*_1_; and (**d**) parameter optimization of *D*_1_.

**Figure 8:**
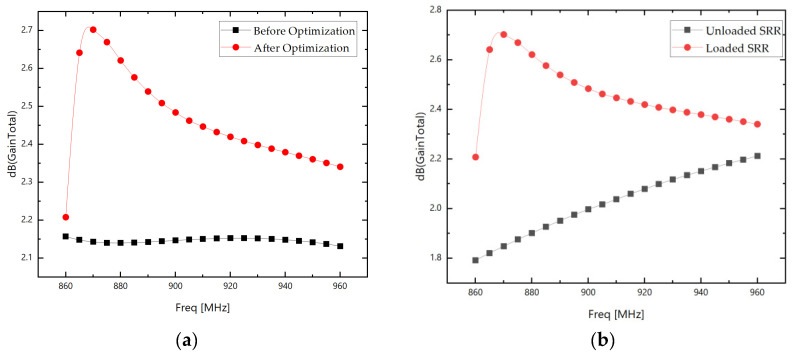
(**a**) Comparison chart of gain before and after optimization; (**b**) comparison chart of gain before and after loading the optimized SRR.

**Figure 9:**
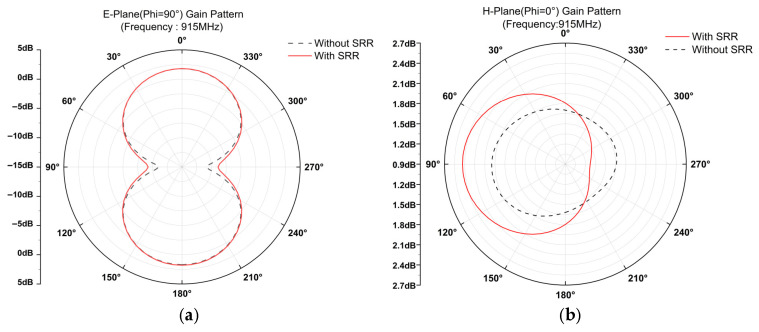
Comparison of two-dimensional E/H-plane radiation patterns at 915 MHz before and after SRR loading. (**a**) Comparison of E-plane radiation before and after loading at 915 MHz; (**b**) comparison of H-plane radiation before and after loading at 915 MHz.

As shown in Figure 6, the gain improvement of the RFID antenna after loading the Two-element SRR resonant superstrate unit is not significant compared to the unloaded state. To further enhance the gain boost from the SRR, optimization of the unit’s dimensions is required. The specific optimization parameters are listed in Table 3:

The brief optimization of individual parameters of ring *C*_1_ is shown in Figure 7 (for the optimization of other parameters and the parameters of ring *C*_2_, please refer to Appendix A):

The final parameter values after the joint optimization of multiple size parameters and the original parameter values are shown in Table 4:

Finally, the gain comparison diagram before and after loading the SRR antenna parameter optimization is shown. The gain comparison between the SRR loaded after optimization and the antenna without SRR loading is shown in Figure 8:

It can be observed that the antenna gain has improved to some extent before and after loading the SRR. The maximum gain increase within the 860 MHz–960 MHz frequency band is approximately 0.85 dB; the average gain increase is approximately 0.44 dB. The final two-dimensional E/H plane radiation comparison at the center frequency of 915 MHz before and after loading the SRR is shown in Figure 9:

The SRR loading achieves complementary optimization of antenna performance: On the E-plane, the maximum gain increases slightly from 1.79 dB to 1.81 dB after SRR loading, while maintaining stable main radiation performance. The minimum gain significantly improved from −11.09 dB to −9.27 dB, narrowing the gain fluctuation range by 1.8 dB. This effectively mitigates energy attenuation in non-main directions and reduces “deep valleys” in the radiation pattern. For the H-plane, the maximum gain increased from 2.00 dB to 2.43 dB, a rise of 0.43 dB. Energy concentration in the main radiation direction markedly strengthened, with no pattern distortion or abnormal side lobes observed. Evidently, the SRR optimizes E-plane radiation uniformity and enhances H-plane main radiation efficiency without compromising the antenna’s original directivity. This comprehensively improves antenna radiation performance, validating its effectiveness and engineering application potential in antenna structural optimization.

### 3.4. Physical Testing

**Figure 10:**
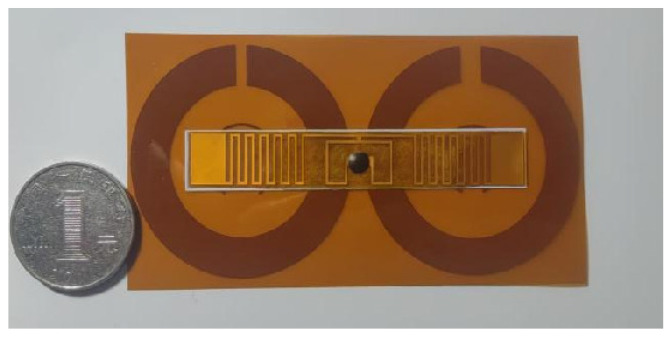
A physical picture of the RFID tag antenna loaded with SRR.

**Figure 11:**
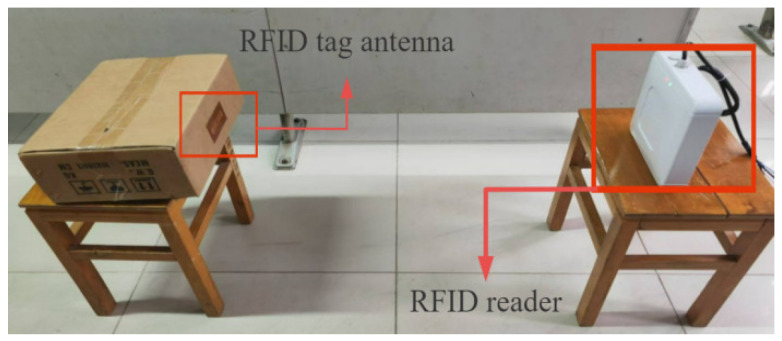
Physical test schematic diagram.

The RFID tag antenna with the SRR loaded is shown in Figure 10. Physical testing is illustrated in Figure 11, where the RFID tag antenna and the RFID tag antenna with the Two-element SRR resonant superstrate loaded were successively affixed to the same position on a delivery box. The forward reading distance was then directly measured using an RFID reader.

**Figure 12:**
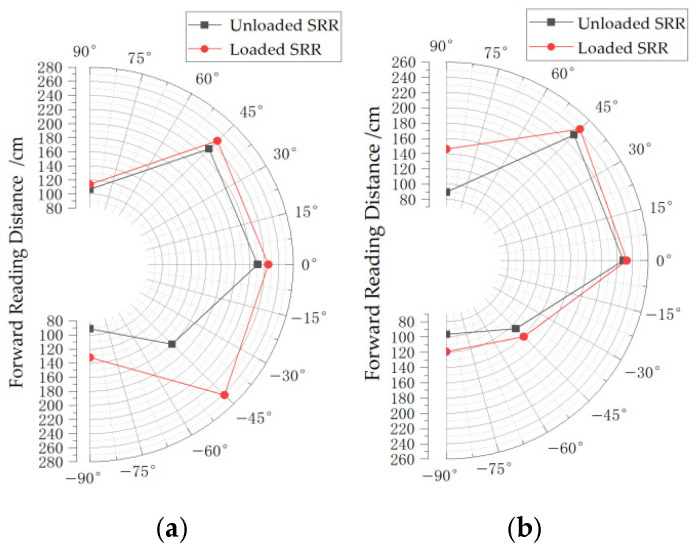

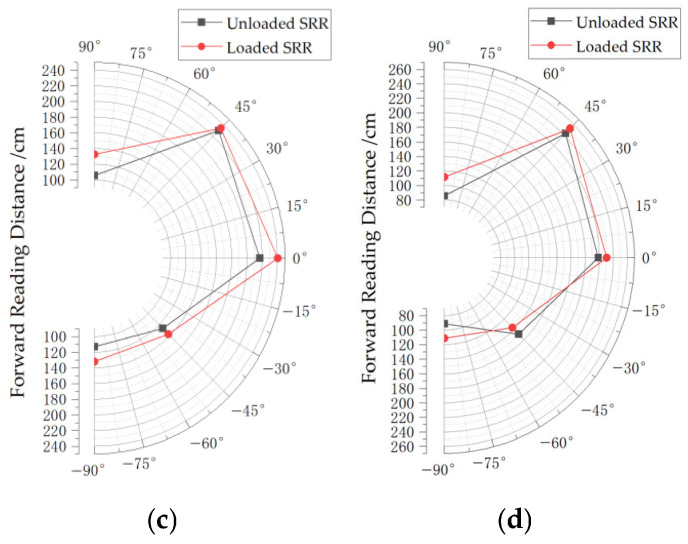
(**a**) Comparison of the actual maximum forward read distance before and after loading SRR at the frequency point of 921.125 MHz; (**b**) comparison of the actual maximum forward read distance before and after loading SRR at the frequency point of 922.125 MHz; (**c**) comparison of the actual maximum forward read distance before and after loading SRR at the frequency point of 923.125 MHz; and (**d**) comparison of the actual maximum forward read distance before and after loading SRR at the frequency point of 924.125 MHz.

As the specific usage frequency of RFID technology in the 900 MHz band stipulated in China is 920 to 925 MHz, the following only shows the maximum forward reading distance of the RFID tag at different angles facing the reader when the reader’s transmission frequency is between 921.125 MHz and 924.125 MHz with an interval of 1 MHz. As shown in Figure 11, the angle at which the RFID tag antenna faces the reader is supposed to be 0°. Table 5 and Figure 12 compare the actual maximum forward reading distances before and after loading the SRR at each frequency point:

The chart demonstrates that incorporating the SRR structure enhances the antenna’s reading distance. The maximum improvement reaches 1.02 m, approximately 62.1% of the original antenna’s average reading distance. The average improvement is about 21.8 cm, representing 13.8% of the original antenna’s average reading distance. The limited improvement observed in physical tests with the SRR structure stems from several factors: First, the reader’s operating frequency is constrained between 920 and 925 MHz, which is not the frequency band where the SRR structure achieves its highest gain enhancement. Additionally, during measurements, the antenna was affixed to the parcel box and may not have been perfectly flat, causing some bending that could have impacted performance gains.

Furthermore, the antenna exhibits minimal gain improvement at the −45° position across all frequency points. A slight decrease is even observed at −45° for 924.125 MHz, potentially due to coupling efficiency reduction caused by mode distortion, which affects the antenna’s read range.

## 4. Conclusions and Discussion

This paper proposes an SRR resonant superstrate-enhanced UHF RFID tag antenna technology. A folded dipole antenna gain-enhancing SRR resonant superstrate was designed, and its optimal structural parameters were obtained using a multi-parameter joint optimization method. After prototyping the RFID antenna and the Two-element SRR resonant superstrate and conducting comparative performance tests, the following conclusions were drawn:

1. The Two-element SRR resonant superstrate loading enhances the gain of the UHF RFID dipole tag antenna to a certain extent. The maximum gain improvement within the 860 MHz–960 MHz frequency band reaches approximately 0.85 dB, with an average gain increase of about 0.44 dB;
2. The UHF RFID dipole tag antenna equipped with the Two-element SRR resonant superstrate demonstrated an enhanced maximum read range. Within the 921–925 MHz band, the maximum range improvement reached 1.02 m, equivalent to 62.1% of the original antenna’s average read distance. The average range improvement was approximately 21.8 cm, representing 13.8% of the original antenna’s average read distance.

To the best of the authors’ knowledge, this work is the first to demonstrate utilizing a special structure with dual-element SRR coupling to realize local magnetic field enhancement and near-field coupling optimization for RFID tag antennas. This approach enhances the radiation gain and directivity of RFID tag antennas, ultimately improving their overall gain and maximum forward reading distance. Compared to conventional gain strategies based on standard antennas or traditional metasurface elements, this work employs directional amplification and resonant coupling control of the near-field magnetic field via a Two-element SRR resonant superstrate. This approach achieves higher port gain without significantly increasing loss or volume, demonstrating a novel design pathway. This work establishes a novel design paradigm for SRR-RFID antenna coupling utilizing SRRs as magnetic response units for near-field directional control, enabling the realization of efficient, compact, and reproducibly manufacturable high-gain tag antennas with significant application potential.

This technology can be applied to precision logistics tracking (such as the management of high-value goods or luxury items), critical asset monitoring (such as medical equipment or military hardware), and automated inventory systems within smart factories, ensuring stable and efficient long-range identification in complex environments.

## Figures and Tables

**Table 1:** RFID dipole tag antenna size.

Parameter	Value/mm	Parameter	Value/mm
*W*	0.725	*W*_4_	7
*W*_0_	3.275	*L*_1_	8.8
*W*_1_	4.2	*a*	71
*W*_2_	0.83	*b*	11
*W*_3_	0.7	*h*	0.13

**Table 2:** The unit size of SRR.

Parameter	Value/mm	Parameter	Value/mm
*R*_0*C*1_	23	*R*_0*C*2_	23
*R*_1*C*1_	7.5	*R*_1*C*2_	7.5
*L*_1_	1.35	*L*_2_	1.35
*W*_1_	7	*W*_2_	7
*D*_1_	4.5	*D*_2_	4.5
*W*_3_	1	*W*_4_	1
*D*_3_	4.5	*D*_4_	4.5

**Table 3:** Optimization of size parameters of the Two-element SRR resonant superstrate.

Parameter	Original Value/mm	The Scope of Optimization/mm	Step/mm
*R*_0*C*1_	23.0	23.2–22.6	0.1
*R*_1*C*1_	7.50	6.75–7.75	0.25
*L*_1_	1.350	1.25–1.40	0.025
*W*_1_	7.0	6.8–7.6	0.2
*D*_1_	4.50	2–5	0.1
*W*_3_	1.0	0.4–1.2	0.1
*D*_3_	4.50	3–6	0.1
*R*_0*C*2_	23.0	23.2–22.6	0.1
*R*_1*C*2_	7.50	7.0–8.0	0.1
*L*_2_	1.350	1.25–1.40	0.025
*W*_2_	7.0	5–9	0.1
*D*_2_	4.50	2–5	0.1
*W*_4_	1.0	0.4–1.2	0.1
*D*_4_	4.50	4–8	0.1

**Table 4:** The parameter values before and after the optimization of the SRR resonant superstructure.

Parameter	Pre-Optimization/mm	Post-Optimization/mm	Parameter	Pre-Optimization/mm	Post-Optimization/mm
*R*_0*C*1_	23	23	*R*_0*C*2_	23	23
*R*_1*C*1_	7.5	7.0	*R*_1*C*2_	7.5	7.5
*L*_1_	1.35	1.35	*L*_2_	1.35	1.3
*W*_1_	7	7.2	*W*_2_	7	8
*D*_1_	4.5	4.5	*D*_2_	4.5	2.25
*W*_3_	1	0.6	*W*_4_	1	0.5
*D*_3_	4.5	4.25	*D*_4_	4.5	7

**Table 5:** Comparison of the Maximum Forward Reading Distance Before and After Loading at Each Frequency Point.

Frequency/MHz	Angle (°)	Pre-Load Read Distance/cm	Post-Loading Read Distance/cm
921.125	−90	91.3	132
	−45	160	262
	0	231	245.8
	45	232	248.25
	90	106.75	113.75
922.125	−90	96.6	119.4
	−45	126.3	141
	0	228	232.5
	45	232.6	243.3
	90	89.6	146
923.125	−90	113	132
	−45	127	137.25
	0	217	240.6
	45	230.3	234.5
	90	105.5	132.5
924.125	−90	91.3	111.25
	−45	149.5	136.6
	0	218.6	230.51
	45	243.3	252.6
	90	85.6	111.75

**Figure A1:**
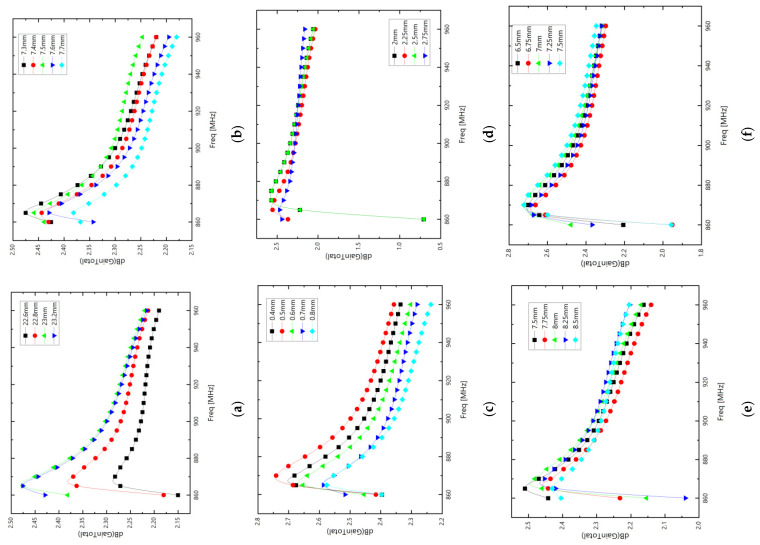

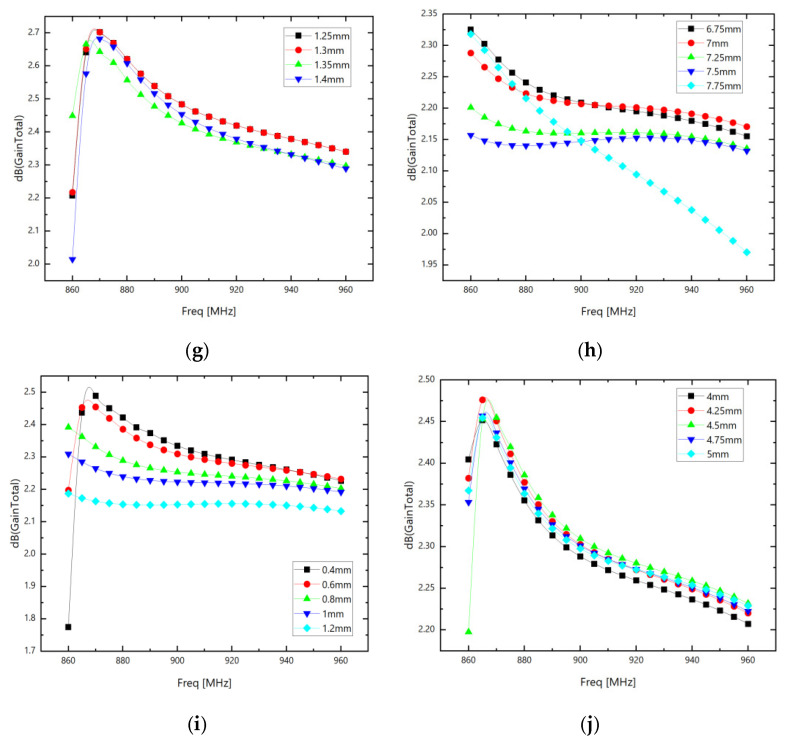
(**a**) Parameter optimization of *R*_0*C*2_; (**b**) parameter optimization of *R*_1*C*2_; (**c**) parameter optimization of *D*_2_; (**d**) parameter optimization of *W*_2_; (**e**) parameter optimization of *W*_4_; (**f**) parameter optimization of *D*_4_; (**g**) parameter optimization of *L*_2_; (**h**) parameter optimization of *R*_1*C*1_; (**i**) parameter optimization of *W*_3_; and (**j**) parameter optimization of *D*_3_.

## Data Availability

The data are available upon request from the corresponding author.

## Appendix A

The undisplayed parameters of ring *C*_1_ and the brief optimization of each parameter of *C*_2_ are as follows:
